# A Fusion-Inhibiting Peptide against Rift Valley Fever Virus Inhibits Multiple, Diverse Viruses

**DOI:** 10.1371/journal.pntd.0002430

**Published:** 2013-09-12

**Authors:** Jeffrey W. Koehler, Jeffrey M. Smith, Daniel R. Ripoll, Kristin W. Spik, Shannon L. Taylor, Catherine V. Badger, Rebecca J. Grant, Monica M. Ogg, Anders Wallqvist, Mary C. Guttieri, Robert F. Garry, Connie S. Schmaljohn

**Affiliations:** 1 United States Army Medical Research Institute of Infectious Diseases, Virology Division, Fort Detrick, Maryland, United States of America; 2 DoD Biotechnology High Performance Computing Software Applications Institute, Telemedicine and Advanced Technology Research Center, United States Army Medical Research and Materiel Command, Fort Detrick, Maryland, United States of America; 3 Tulane University, Department of Microbiology and Immunology, New Orleans, Louisiana, United States of America; Centers for Disease Control and Prevention, United States of America

## Abstract

For enveloped viruses, fusion of the viral envelope with a cellular membrane is critical for a productive infection to occur. This fusion process is mediated by at least three classes of fusion proteins (Class I, II, and III) based on the protein sequence and structure. For Rift Valley fever virus (RVFV), the glycoprotein Gc (Class II fusion protein) mediates this fusion event following entry into the endocytic pathway, allowing the viral genome access to the cell cytoplasm. Here, we show that peptides analogous to the RVFV Gc stem region inhibited RVFV infectivity in cell culture by inhibiting the fusion process. Further, we show that infectivity can be inhibited for diverse, unrelated RNA viruses that have Class I (Ebola virus), Class II (Andes virus), or Class III (vesicular stomatitis virus) fusion proteins using this single peptide. Our findings are consistent with an inhibition mechanism similar to that proposed for stem peptide fusion inhibitors of dengue virus in which the RVFV inhibitory peptide first binds to both the virion and cell membranes, allowing it to traffic with the virus into the endocytic pathway. Upon acidification and rearrangement of Gc, the peptide is then able to specifically bind to Gc and prevent fusion of the viral and endocytic membranes, thus inhibiting viral infection. These results could provide novel insights into conserved features among the three classes of viral fusion proteins and offer direction for the future development of broadly active fusion inhibitors.

## Author Summary

Entry into a cell is an essential stage of the viral replication cycle. Enveloped viruses require fusion of viral and cellular membranes for the viral genome to enter the cell cytoplasm. This entry is mediated by a viral fusion protein. Here, we synthesized peptides based on the Rift Valley fever virus (RVFV) fusion protein stem region and tested the peptides for their ability to prevent RVFV infection of cell cultures. We found that one of these peptides was able to inhibit RVFV infectivity by preventing the fusion process and that this peptide had broad activity against other RNA viruses including Ebola, Andes, and vesicular stomatitis viruses.

## Introduction

Rift Valley fever (RVF) is a disease of major public health and economic concern, affecting humans and livestock throughout Africa [1]–[6] and the Arabian Peninsula [7]. The etiological agent of this zoonosis, Rift Valley fever virus (RVFV), is an arbovirus belonging to the *Phlebovirus* genus in the family *Bunyaviridae*. RVFV infection is severe in animals, especially sheep, cattle, and goats, resulting in high mortality rates in newborns and near 100% abortion rates in pregnant animals. In humans, infection is usually self-limiting, but a small percent of cases (1–2%) can progress to severe hepatitis with hemorrhagic manifestations. In addition, retinal inflammation can lead to permanent vision loss in about 1–10% of infected patients [6].

Like other bunyaviruses, RVFV is an enveloped RNA virus containing three genome segments. The large (L) segment encodes the viral polymerase, the medium (M) segment the glycoproteins, Gn and Gc, and two non-structural proteins, and the small (S) segment the nucleocapsid protein, N, and the nonstructural protein NSs. RVFV entry into permissive cells is mediated by Gn and Gc, with Gc being a class II fusion protein [8], [9] that uses a low pH-dependent fusion mechanism following endocytosis [10]. While little is known about the fusion process of RVFV, the functional aspects of other class II fusion proteins have been well characterized [11]. For example, the flavivirus fusion protein, E, binds to a cellular receptor, and the virus enters cells by endocytosis. Acidification of endocytic vesicles results in a low-pH dependent conformational shift in E, rearranging from a dimer to a trimer [12] and inserting a previously hidden fusion peptide into the target cellular membrane [13]–[16]. A second rearrangement of the trimer pulls the viral and cellular membranes into close proximity to allow membrane disruption and fusion to occur [14]. Based on structural modeling, the hydrophobic residues of the stem region N-terminal to the transmembrane domain of E likely moves through a groove formed between domains II in the E trimer during this second rearrangement; as the stem travels through this groove, domain I remains (with the fusion loop inserted into the endocytic membrane) while the C-terminus of domain III is pulled toward the host membrane domain [13].

The large conformational rearrangements that take place during the fusion process present potential opportunities to disrupt early stages in viral replication and prevent a productive infection [11], [13]. Targeting this viral entry process with inhibitory peptides has proven successful with multiple viruses including dengue virus (DENV) [17]–[19], SARS coronavirus [20], and most notably, HIV-1 [21], [22]. The mechanism of action for various inhibitory peptides appears to differ depending on the region of the fusion protein used to design the peptide. For DENV, peptides analogous to the hinge region of domain II or the beta sheet interaction between domains I and II is thought to prematurely trigger a rearrangement of the viral glycoproteins and thus interfere with virion binding to the target cell [19]. In contrast, peptides homologous to the hydrophobic DENV stem region of the fusion protein interfere with fusion of the viral and cellular membranes [18]. Data suggest that this stem peptide interferes with the movement of the viral stem region along domain II, preventing the two membranes from coming in close enough proximity to fuse [18].

Another mechanism of entry inhibition was described for HIV-1 involving gp41 rearrangement in response to gp120 binding of CD4 and either the co-receptor CCR5 or CXCR4. This rearrangement exposes the C- and N-terminal heptad repeat domains which, following the second gp41 rearrangement, form a 6-helix bundle in the post-fusion state [23]. Peptides analogous to the C-terminal heptad repeat bind to the exposed N-terminal heptad repeat when theses domains are exposed, preventing completion of the second rearrangement and formation of the 6-helix bundle [24].

In this report we describe the design and evaluation of peptides based on RVFV's fusion protein, Gc. We demonstrate that one of the peptides has broad spectrum activity against viruses with Class I, II, and III fusion mechanisms, and we present a model for the potential mechanism of action of this peptide for inhibiting infections.

## Materials and Methods

### Identification and synthesis of potential inhibitory peptides

The RVFV Gc amino acid sequence (GenBank P03518) was analyzed for a positive Wimley-White interfacial hydrophobicity score (WWIHS), as previously described [17], [20], using the program Membrane Protein eXplorer [25]. Peptides were generated based on a positive WWIHS and protein domain consideration, and regions selected for peptide generation include Gc domains IIa, IIb, III, and the stem region (Table 1). Control, scrambled peptides (designated with a –sc) were generated by randomly assigning amino acid positions for each amino acid in the experimental peptide. Peptides were synthesized by a solid-phase conventional N-a-9-flurenylmethyloxcarbonyl chemistry and purified by reverse-phase high performance liquid chromatography to greater than 95% purity (Bio-synthesis, Inc., Lewisville, TX). Lyophilized peptides were initially resuspended in 1,1,1,3,3,3-hexafluoro-2-propanol (Sigma-Aldrich, St. Louis, MO) overnight and dried in a vacuum centrifuge. Stock solutions were generated by resuspending all peptides in 20–30% dimethyl sulfoxide (DMSO) (Sigma-Aldrich, St. Louis, MO) and water (Life Technologies, Grand Island, NY). Peptide concentrations were determined by measuring the absorbance of aromatic amino acid side chains at 280 nm using a Nanodrop spectrophotometer (Thermo Scientific, Wilmington, DE). Working stocks of peptides were generated by adding stock peptide to complete cell culture medium (see below).

**Table 1:** Peptide amino acid sequences analogous to the domain and location within RVFV Gc.

peptide	amino acid sequence	domain	location
RVFV-1	YWTGSISPKCLSSRRCHLV	IIa	72–90
RVFV-2	WGCGCFNVNPSCLFVHTYL	IIa (fusion peptide)	131–149
RVFV-3	LGASSSRFTNWGSVSLSLD	IIb	185–203
RVFV-4	FVGAAVSCDAAFLNLTGCY	III	332–350
RVFV-5	WNFFDWFSGLMSWFGGPLKLY	stem	450–470
RVFV-6	WNFFDWFSGLMSWFGGPLK	stem	450–468
RVFV-7	WNFFDWFSGLMSWFGGPLKTI	stem	450–470
RVFV-8	SWNFFDWFSGLMSWFGGPLK	stem	449–468
RVFV-9	SGSWNFFDWFSGLMSWFGG	stem	447–465
RVFV-10	SGSWNFFDWFSGLMSWFGGPL	stem	447–467
RVFV-6sc	MFLGWSFDFGSLWGNKPWF	stem	450–468
RVFV-10sc	WSSGLPFGNFGLSWFDMGFWS	stem	447–467

### Viruses, cells, and media

RVFV vaccine strain MP12 [26], [27], the wild-type RVFV-ZH501 [4], [28], ANDV 808034 [29], and a green-fluorescent protein (GFP) tagged Zaire Ebolavirus, EboZ-eGFP [30], [31] were used in the assays. EboZ-eGFP was kindly provided by Dr. Jonathan Towner, Centers for Disease Control and Prevention (Atlanta, GA). These virus stocks are maintained at the U.S. Army Medical Research Institute of Infectious Diseases (USAMRIID), and IRB approval is not required for use. The pseudotyped viruses RVF-VSV-luc and VSV-luc were kindly provided by Dr. Robert Doms at the University of Pennsylvania (Wojcechowskyj et al., unpublished data). Vero E6 cells were maintained at 37°C with 5% CO_2_ in complete medium (cEMEM) consisting of Eagle's minimum essential medium (EMEM, Lonza, Basel, Switzerland) supplemented with 10% (v/v) fetal bovine serum (Life Technologies, Grand Island, NY), 100 U/ml penicillin G (Life Technologies), and 100 mg/ml streptomycin (Life Technologies).

### Virus inhibition assays

For the plaque-reduction assays, 6-well plates of confluent Vero E6 cells were infected with 50–75 plaque forming units (pfu) of virus that was pre-incubated with or without peptide in cEMEM for 1 h at 37°C. Virus was allowed to adsorb for 1 h at 37°C after which the monolayers were washed once with phosphate buffered saline (PBS, Life Technologies, Grand Island, NY) and overlaid with EBME (Life Technologies, Grand Island, NY) supplemented with 10% FBS, 1% non-essential amino acids, 4% L-glutamine (Life Technologies, Grand Island, NY), 100 U/ml penicillin G, 100 mg/ml streptomycin, and 1× Fungizone (Life Technologies, Grand Island, NY) containing 0.6% (w/v) SeaKem ME agarose (Lonza, Basel, Switzerland). Cells were incubated at 37°C with 5% (v/v) CO_2_ for 3 days (RVFV) or 7 days (ANDV), and a secondary overlay containing EBME supplemented with 10% FBS, 100 U/ml penicillin G, 100 mg/ml streptomycin, 1× Fungizone, and 5% neutral red (Life Technologies, Grand Island, NY) was added. Plaques were subsequently counted over 2 days starting the following day for RVFV and 3 days following the addition of the secondary overlay for ANDV.

For the EboZ-eGFP and pseudotyped infections, signal-optimized concentrations of virus [31] were incubated with a dilution series of each peptide diluted in cEMEM. After a 1 h incubation, media were removed from the 96-well plates of confluent Vero E6 cells, and virus/peptide was added to triplicate wells. After a 1 h incubation, the inocula were removed, the cells washed once with PBS, and fresh media added. For EboZ-eGFP, levels of GFP were measured 48 h after-infection. For the pseudotyped viruses, luciferase activity was measured the following day using the *Renilla* Luciferase Assay System (Promega, Madison, WI) according to the manufacturer's instructions.

### MTT toxicity assay

Peptide toxicity was assessed using the MTT cell proliferation assay (ATCC, Manassas, VA) according to the manufacturer's instructions. Briefly, Vero E6 cells were incubated with 100 µl cEMEM containing each peptide for approximately 18 h before the addition of tetrazolium salt (MTT). This salt is reduced in metabolically active cells, forming crystals which are solubilized by detergent. Absorbance was read at 570 nm with a 96-well plate spectrophotometer (Promega/Turner Biosystems, Madison, WI).

### Peptide-cell binding assays

To assess peptide binding to cells, a C-terminal biotin conjugated RVFV-6 peptide and a biotin-conjugated RVFV-6 scrambled peptide were synthesized (Bio-synthesis, Inc., Lewisville, TX). An immunofluorescence assay was developed to detect peptide binding to Vero E6 cells. Cells were transfected with a plasmid containing a codon-optimized RVFV-ZH548 GnGc expression construct. Cells were incubated with 25 µM peptide in chamber slides for either 30 seconds or 1 h. Following a 1 hour incubation, cells were washed extensively with PBS before fixing in 10% buffered formalin (Fisher Scientific, Pittsburg, PA) for 15 min. An anti-biotin antibody conjugated to a Texas Red fluorophore (Abcam, Cambridge, MA) was incubated with the cells for 1 h. After washing with PBS, cells were mounted with a DAPI-containing mounting medium (Life Technologies, Grand Island, NY) and observed under a microscope. Pictures were taken and merged to depict peptide binding (red) and nuclei (blue).

Electron microscopy was conducted to visualize peptide binding to Vero E6 cells treated with and without RVFV-6 peptide. For immunogold labeling, cell monolayers were briefly pre-fixed in 0.2% paraformaldehyde (E.M. Sciences, Warrenton, PA) at room temperature. After this brief fixation, the cells were washed in PBS and incubated with goat anti-biotin 15 nm IgG Gold antibody (Ted Pella, Redding, CA) for 2 h at room temperature. After the wash steps, the attached cells were fixed in with 2.5% glutaraldehyde (E.M. Sciences), scraped, and pelleted by centrifugation. Cell pellets were minced into small pieces, washed in Millonig's sodium phosphate buffer (Tousimis Research, Rockville, MD), and stored overnight at 4°C. The samples were then post-fixed in 1.0% osmium tetroxide (E.M. Sciences), *en bloc* stained with 2.0% aqueous uranyl acetate, dehydrated in a series of graded ethanols, and infiltrated and embedded in DER 736 plastic resin (Tousimis Research). After polymerization for 48 h at 70°C, blocks from each sample were ultra-thin sectioned using Leica UC7 Ultramicrotome (Leica Microsystems, Buffalo Grove, IL). Thin sections 60 to 80 nm in thickness were collected from each sample and mounted onto 300 mesh copper grids. The grids from each sectioned block were then post-stained with Reynold's lead citrate and subsequently viewed in a Tecnai Spirit Twin transmission electron microscope, operating at 80 kV.

### Peptide-virion binding assay

In order to address the mechanics of peptide inhibition of the virus, a binding assay was developed. Twenty-five µl biotin-conjugated RVFV-6 or biotin-conjugated RVFV-6 scrambled peptide was incubated with streptavidin magnetic beads (Life Technologies, Grand Island, NY). After peptide binding to the beads, unbound peptide was washed away with Tris-buffered saline (TBS, Sigma-Aldrich, St. Louis, MO). RVFV-MP12 diluted in cEMEM was added to the beads for 1 h at 37°C, allowing for peptide-virion binding. After the 1 h, the beads were washed with Tris-buffered saline (TBS) and treated in one of three conditions: 1) virus bound to beads were lysed using 1% Triton X-100 (Sigma-Aldrich, St. Louis, MO), 2) virus bound to the beads were treated with Earl's salt solution containing 20 mM HEPES and 20 mM MES, pH 5.2 (low pH medium) for 15 min to trigger pH-induced glycoprotein rearrangements prior to being lysed, or 3) virus was not pH treated and not lysed. The magnetic beads were washed with TBS (or low pH medium for the pH treated beads) to remove unbound virus, and SDS-PAGE loading buffer (Life Technologies, Grand Island, NY) was added to the beads. After a 5 min incubation at 70°C, samples were resolved on a SDS-PAGE gel. The resolved proteins were transferred to a nitrocellulose blot, blocked with 5% Difco (Becton-Dickenson, Franklin Lakes, NJ) in PBS (block), and incubated with a 1∶1000 dilution in block of the mouse anti-RVFV Gc antibody 4D4 [32]. After three washes with PBS containing 0.05% Tween-20 (PBST, Sigma-Aldrich, St. Louis, MO), a secondary horse radish peroxidase conjugated goat anti-mouse antibody (Santa Cruz Biotechnology, Santa Cruz, CA) diluted 1∶2500 dilution in block was added for 1 h. The blot was washed in PBST and imaged using a camera system (G-box, Syngene, Frederick, MD).

### Virion-cell binding assay

A probe-based, real-time RT-PCR assay was used as previously described for RVFV [33] and EBOV [34] to detect the relative amount of virus present in a sample. Two dilutions of RVFV or EboZ-eGFP, 10^4^ and 10^5^ pfu) were pre-treated with 25 µM peptide for 1 h before infecting a monolayer of Vero E6 cells. One hour post-infection, cells were washed extensively with phosphate buffered saline (PBS) to remove unbound virus, and total RNA was extracted using TRIzol (Life Technologies, Grand Island, NY) according to the manufacturer's instructions. Equal amounts of RNA were used in the real-time RT-PCR assay as previously described using the Power SYBR Green RNA-to-Ct 1-Step Kit (Applied Biosystems/Life Technologies, Grand Island, NY) on a Bio-Rad CFX96 real-time instrument (Bio-Rad, Hercules, CA).

### Cell-cell fusion assay

A plasmid based cell-cell fusion assay was developed similar to the alphavirus replicon-based system described previously [10] to assess if RVFV-6 inhibits the fusion process. Codon-optimized RVFV-ZH548 glycoproteins GnGc as well as codon-optimized T7 polymerase were previously cloned into the mammalian dual-expression vector pBud-CE4.1 (Life Technologies, Grand Island, NY) to create the plasmid pBud-CE4.1-RVFV548-GnGc-T7-opti. Vero E6 cells in a 6-well plate were transfected using Fugene HD Transfection Reagent (Promega, Madison, WI) with pBud-CE4.1-RVFV548-GnGc-T7-opti or a mammalian expression plasmid containing a VSV G expression cassette (pVSV-G), kindly provided by Dr. Robert Doms. Approximately 18 h later, the transfected cells were harvested using trypsin/EDTA (Life Technologies, Grand Island, NY) and seeded onto wells of an 8-well chamber slide (Lab-Tek II chamber slide RS, Thermo Scientific, Wilmington, DE). The cells transfected with pBud-CE4.1-RVFV548-GnGc-T7-opti were seeded at 1×10^5^ cells/well. Cells transfected with pVSV-G were seeded at 1.25×10^4^ cells/well, and untransfected cells were added to bring the final concentration to 1×10^5^ cells/well. Twenty-four h later, the media were exchanged with cEMEM with or without diluted peptide. Following a 1 hour incubation at 37°C, the cells were treated at low-pH for 15 min with low pH medium. EMEM was added to the wells to raise the pH, and the slides were incubated at 37°C with 5% CO_2_. Five h later, cells were fixed for 7 min with ice-cold methanol and air dried. Cells were stained for 15 min with a 1∶10 dilution of freshly prepared Giemsa stain (Promega, Madison, WI) in water. Slides were air dried, mounted with a DAPI-containing mounting medium, and were observed under a microscope. Statistical significance for differences between the peptide treated and untreated control was determined using a paired, two-tailed t-test using Prism 5 (GraphPad Software, La Jolla, CA).

### Structural models of the envelope proteins

Generation of the 3-D models for the RVFV, VSV, and EBOV fusion proteins from their sequences was carried out by using the Protein Structure Prediction Pipeline (PSPP) [35]. The PSPP uses the program NEST [36] for generating homology models. Two data inputs were used to produce such models: (a) templates and (b) pair-wise alignments. The template files from the experimentally determined structures of glycoprotein C from RVFV [37] (PDB code 4HJC), Semliki Forest virus [38] (PDB code: 1RER) and Venezuelan equine encephalitis virus (VEEV) [39] (PDB code 3J0C) were used to generate models for RVFV; VSV structure (PDB code:2CMZ) [40] was used to generate a complete VSV model of the fusion trimer; and the structures of Zaire Ebola virus (PDB codes: 2EBO [41] and 3CSY [42]) were used to generate a complete model for the GP2 fusion trimer of EBOV. All experimental structures were obtained from the Protein Data Bank (PDB) [43]. Regions of the models for which structural information was not available were built *de novo* based on secondary structure information. Analysis of the final structures was performed with the help of PyMOL Molecular Graphics System (pymol.org)

## Results

### Design of membrane-interacting peptides

RVFV Gc amino acid sequence was analyzed to identify regions of the predicted protein having a positive Wimley-White interfacial hydrophobicity score (WWIHS), indicating a potential to interact with lipid bilayers [25]. Five non-transmembrane domain regions within RVFV Gc were found with significant WWIHS values, and peptides analogous to these five regions were synthesized (RVFV-1, -2, -3, -4, -5; Table 1).

### Identification of inhibitory peptides against RVFV

**Figure 1:**
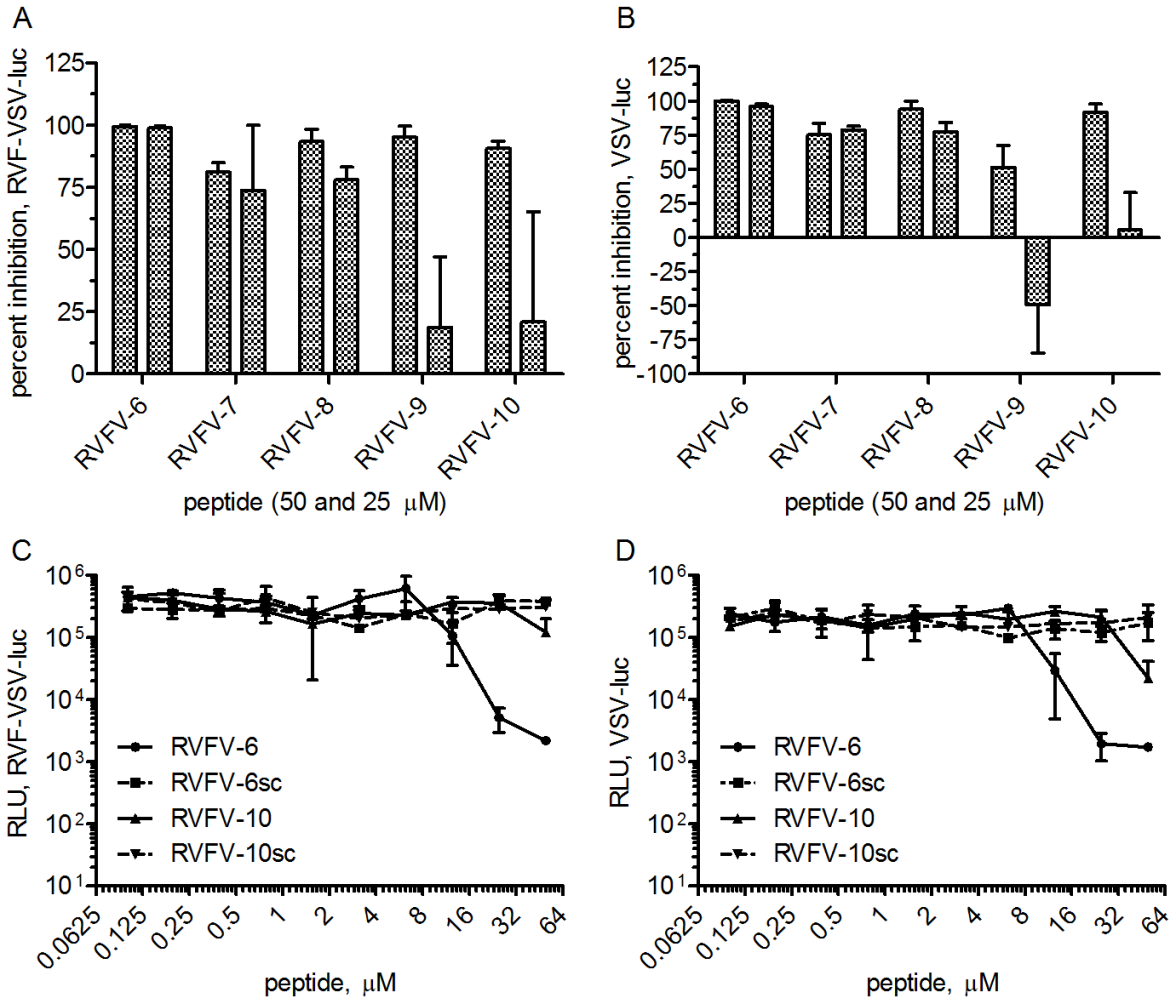
Stem-based peptides inhibit both RVFV and VSV. Peptides were screened for inhibition of the pseudotyped reporter viruses RVF-VSV-luc (A and C) and VSV-luc (B and D). Virus was incubated with peptide RVFV-6, -7, -8, -9, or -10 (A, B) or serial dilutions of RVFV-6, RVFV-10, or the scrambled peptides RVFV-6sc or RVFV-10sc (C, D) prior to infecting a monolayer of Vero E6 cells. Luciferase activity (RLU) was measured approximately 18 h later. Percent inhibition was calculated based on the virus-only controls. Error shown is the standard deviation of the mean. Data are representative of at least 2 experiments.

The initial five RVFV synthetic peptides were evaluated for inhibition of RVFV-MP12 using a plaque-reduction assay. Only RVFV-5 demonstrated inhibition (approximately 30%, data not shown). This peptide is based on the RVFV fusion protein Gc's stem region (amino acids 449–468 of RVFV Gc) N-terminal to the transmembrane domain. Consequently, additional peptides based on RVFV-5 were designed and synthesized, adding or subtracting amino acids from the N- and C-termini of the RVFV-5 peptide analogous region in the pathogenic RVFV-ZH501 viral fusion protein (VFP) stem (GenBank DQ380202). This region included two different amino acids at the C-terminus (Table 1, see RVFV-5 and RVFV-7), and RVFV-8 is analogous to the likely stem sequence (Table 1). These new peptides (RVFV-6, -7, -8, -9, -10, Table 1) were assayed for inhibition using a pseudotyped reporter assay consisting of either a RVFV-pseudotyped VSV-luc reporter virus or a VSV-luc reporter virus. For each virus, the VSV core is tagged with the reporter gene luciferase, and the envelope is composed of either the RVFV glycoproteins Gn and Gc or the VSV G glycoprotein. Each virus was incubated with either 50 µM or 25 µM of each peptide before infecting a monolayer of Vero E6 cells, and luciferase activity was measured as a surrogate for viral replication. Interestingly, all of the peptides inhibited both RVF-VSV-luc and the VSV-luc viruses (Figure 1A and B). Inhibition of VSV-luc was unexpected since the VSV G protein is likely a class III fusion protein [40], [44].

RVFV-6 was selected for further evaluation since RVFV-6 was the strongest inhibitor of both viruses. RVFV-10 was also selected for further evaluation based on the amino acid sequence differences with RVFV-6 and the other peptides (Table 1). Scrambled peptides were made for RVFV-6 and RVFV-10, designated RVFV-6sc and RVFV-10sc (Table 1), by randomly ordering the component amino acids. Inhibition assays were conducted with serial dilutions of the peptides, and luciferase activity was measured (Figure 1C and D). RVFV-6 was the more potent inhibitor of both RVF-VSV-luc and VSV-luc, and the scrambled peptides did not demonstrate inhibition of either virus.

**Figure 2:**
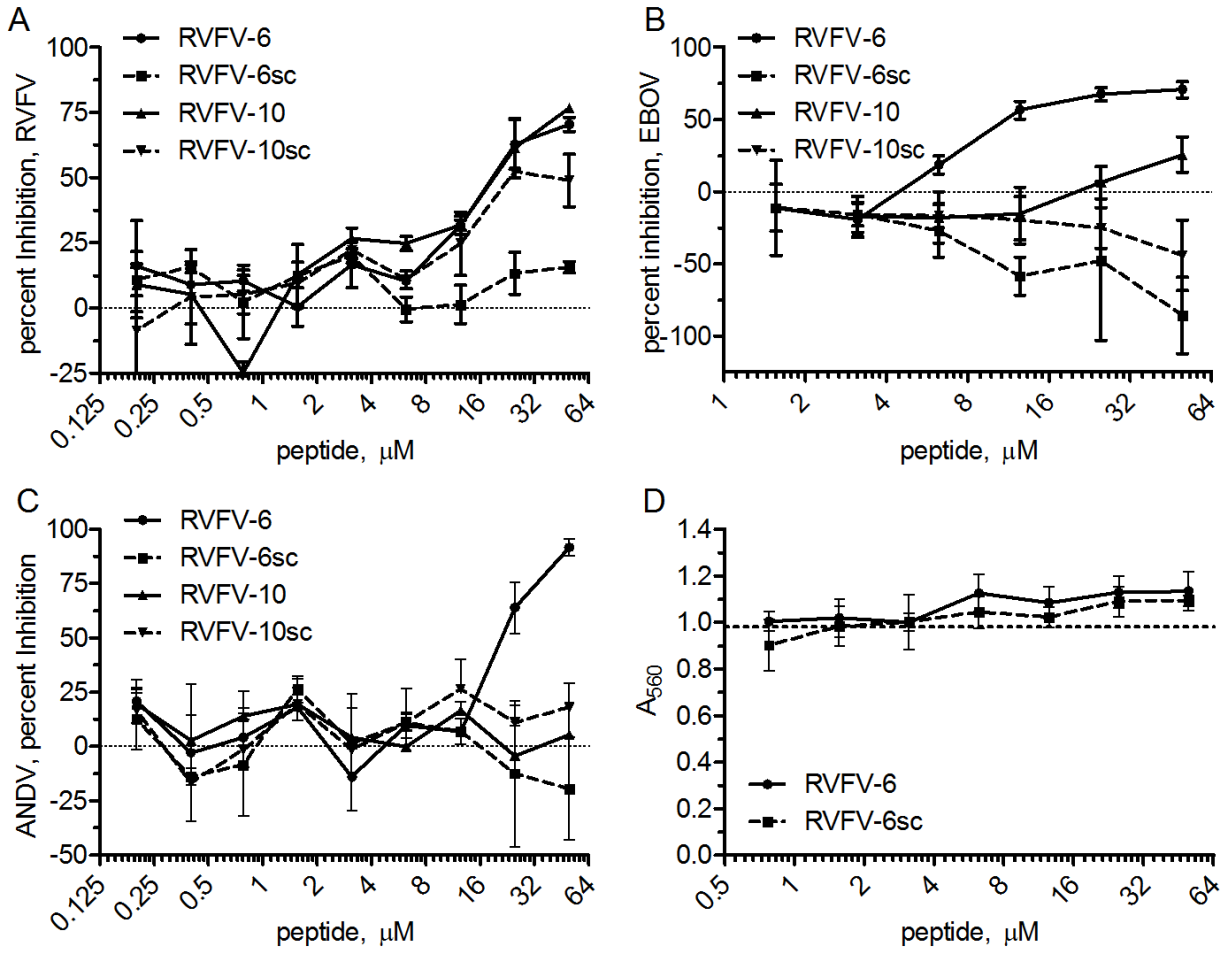
Further characterization of the RVFV stem peptides show broad, non-toxic viral inhibition. Serial dilutions of peptide were incubated with RVFV-ZH501 (A), EboZ-eGFP (B), or ANDV (C), before infecting a monolayer of Vero E6 cells. Percent inhibition was determined for each virus using virus-only controls. (D) MTT toxicity assay results after overnight incubation of Vero E6 cells with RVFV-6 or RVFV-6sc peptides. Absorbance was measured approximately 18 h after adding the diluted peptide in triplicate. The dashed line in (D) represents the average signal generated by the mock treated control cells. Error shown is the standard deviation of the mean. Data are representative of at least 2 experiments.

In order to confirm the inhibition observed with the pseudotyped viruses, RVFV-6 and RVFV-10 were also tested for inhibition of a pathogenic strain of RVFV, RVFV-ZH501, using a plaque-reduction assay. Both RVFV-6 and RVFV-10 strongly inhibited RVFV-ZH501. While RVFV-6sc did not inhibit RVFV-ZH501, RVFV-10sc did (Figure 2A).

### RVFV-6 inhibits additional, diverse viruses

Because both RVFV-6 and RVFV-10 inhibited VSV-luc, we conducted inhibition assays with two other viruses: another member of the *Bunyavirida*e family from a different genus, the hantavirus ANDV, which is also predicted to use class II fusion [45], and a member of the *Filoviridae* family, Ebola virus (EBOV), which has a class I fusion protein [41], [46]. A GFP-tagged EBOV Zaire, EboZ-eGFP, and ANDV were incubated with serial dilutions of RVFV-6, RVFV-10, or the scrambled peptides RVFV-6sc and RVFV-10sc. While both RVFV-6 and, to a lesser extent RVFV-10, inhibited EBOV, only RVFV-6 (Figure 2B and C) strongly inhibited ANDV with nearly 100% reduction in plaques at a 50 µM peptide concentration. Since RVFV-6 inhibited RVFV-ZH501 similar to RVFV-10 (Figure 2A) but inhibited the other viruses (RVF-VSV-luc, VSV-luc, ANDV, and EBOV) better than RVFV-10, RVFV-6 was selected for the subsequent studies. We further evaluated RVFV-6 for inhibition against the alphaviruses Venezuelan (VEEV), western (WEEV), and eastern (EEEV) equine encephalitis viruses using a plaque-reduction assay. No inhibition was observed when using the peptide at 50 µM peptide concentrations for any of these viruses (data not shown).

To rule out the possibility that the decreased virus infectivity was due to peptide toxicity on the Vero E6 cells, we performed a MTT toxicity assay as described in Materials and Methods. Neither RVFV-6 nor RVFV-6sc reduced Vero E6 cell viability at peptide concentrations up to 50 µM (Figure 2D).

### RVFV-6 does not interfere with virion-cell binding

**Figure 3:**
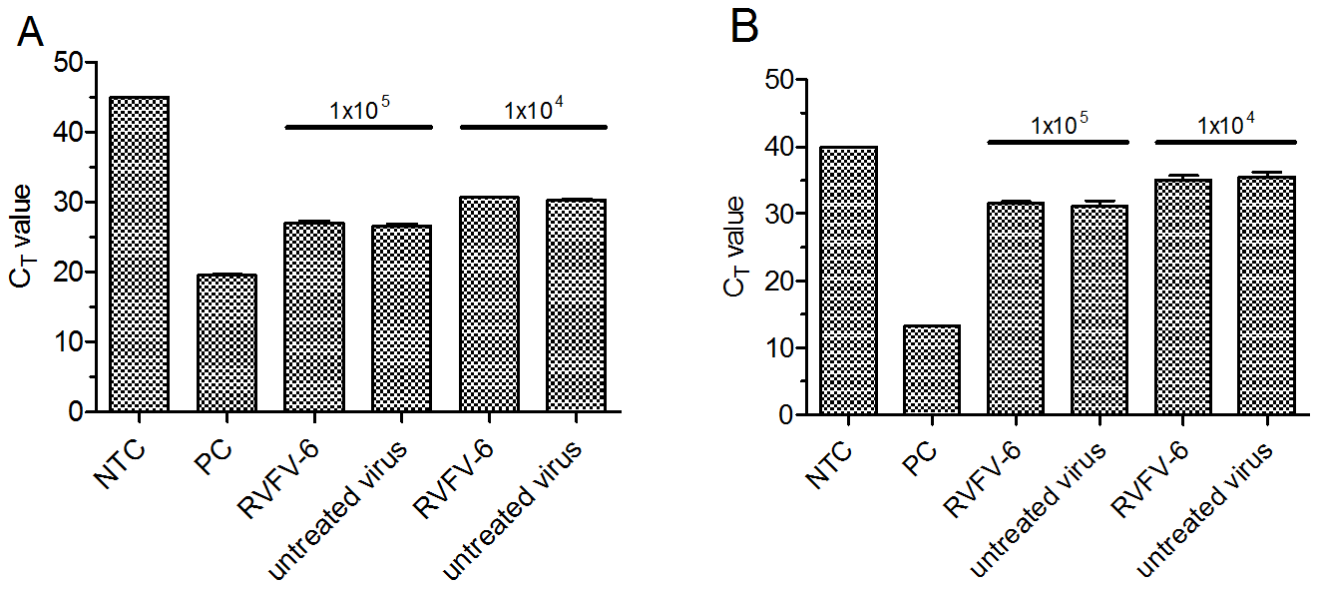
RVFV-6 does not prevent virus from binding to cells. RVFV-MP12 (A) or EboZ-eGFP (B) was incubated with 50 µM RVFV-6 prior to the addition to a confluent monolayer of Vero E6 cells. After a 1 h adsorption, cells were rinsed with PBS, and RNA was harvested using TRIzol. Real time RT-PCR was conducted in triplicate to quantify the relative amount of viral RNA bound to cells, and results are combined from duplicate experiments. NTC is the no template control, PC (positive control) is RNA purified from either RVFV-MP12 or EboZ-eGFP. Untreated virus was mock treated without peptide. Error shown is the standard deviation of the mean.

Because RVFV-6 potently inhibited three diverse viruses that utilize varying fusion mechanisms, we wanted to determine if the peptide was preventing the viruses from binding to permissive cells as has been previously reported for DENV [47]. To evaluate this, RVFV-MP12, which is also inhibited by RVFV-6 (data not shown), or EboZ-eGFP were incubated with or without RVFV-6 prior to addition to Vero E6 cells. Cells were washed with PBS to remove unbound virions, and real-time PCR assays for RVFV [33] or EBOV [34] were used to quantify viral genomes present. If RVFV-6 prevented the viruses from binding to permissive cells, we would expect less viral RNA in RVFV-6 treated cells as compared to untreated cells. For both RVFV and EBOV, there was no measureable difference in the amount of RNA detected in treated and untreated cells (Figure 3), indicating that the peptide did not interfere with viral binding to the target cell.

### RVFV-6 binds to cell membranes nonspecifically but binds specifically to Gc after low pH-triggered rearrangement

**Figure 4:**
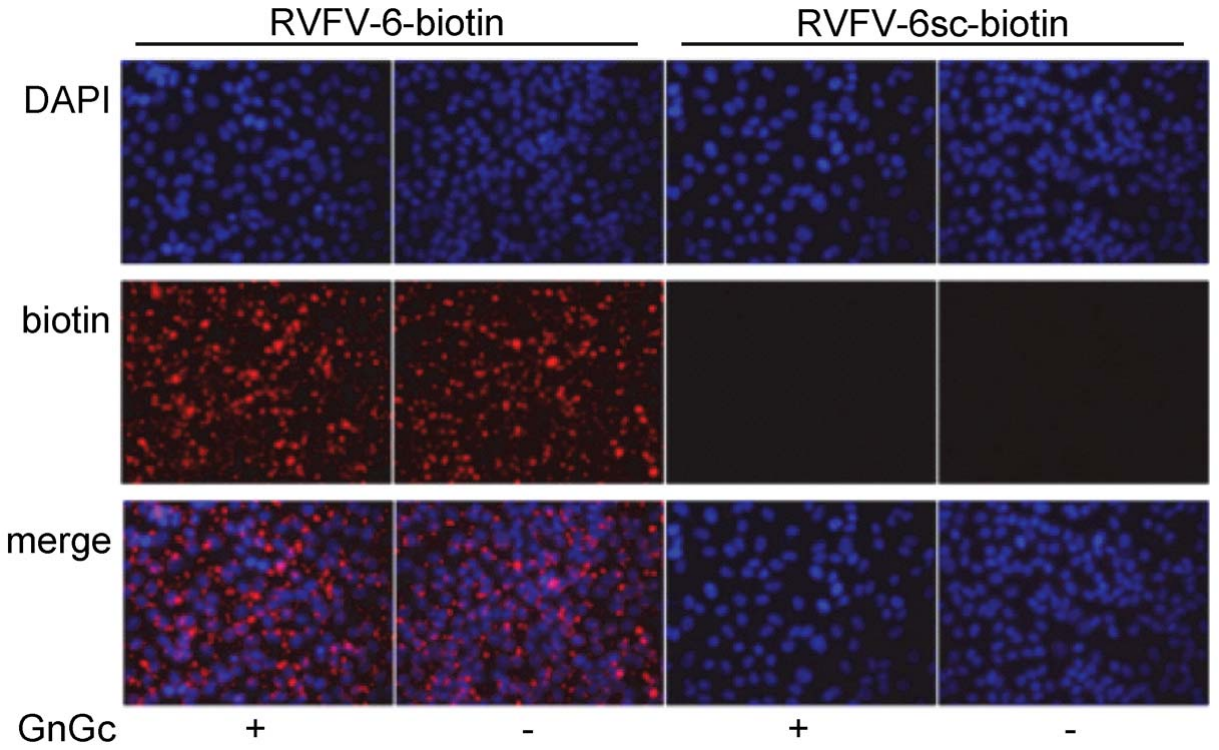
RVFV-6 binds to cells independent of RVFV GnGc expression. Vero E6 cells were transfected with a plasmid expressing RVFV GnGc. Forty-eight h later, either the biotin-labeled RVFV-6 peptide or the biotin-labeled RVFV-6sc peptide was added to the cells followed by washing with PBS. Cells were fixed, and peptide binding was identified using an anti-biotin antibody conjugated to Texas Red. Nuclei were stained with DAPI (blue).

Since RVFV-6 did not interfere with the virus binding to permissive cells, inhibition must have occurred at a later stage of viral entry. To determine if RVFV-6 specifically interacted with RVFV envelope proteins, we transfected cells with a plasmid expressing the RVFV M segment and incubated the cells with either biotin-conjugated RVFV-6 or biotin-conjugated RVFV-6sc. Control cells were mock-transfected. After staining with an anti-biotin antibody, we found that RVFV-6 bound to Vero E6 cells independent of GnGc expression while RVFV-6sc did not bind to either transfected or control cells (Figure 4). The RVFV-6 peptide binding was rapid and could be detected at the earliest measured time (30 seconds, data not shown). The binding could also be visualized by electron microscopy of cells incubated with biotinylated RVFV-6 prior to fixing and staining, with the peptide appearing to form aggregates on the cell surface (Figure S1). These results suggest that the RVFV-6 peptide binds non-specifically to the plasma membrane.

**Figure 5:**
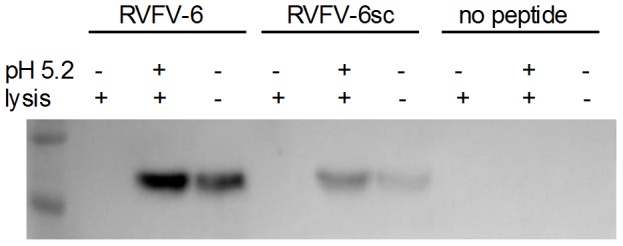
Activation of the viral fusion process is required for RVFV-6 binding to RVFV Gc. Biotin-conjugated RVFV-6, RVFV-6sc, or no peptide was pre-bound to avidin beads before the addition of RVFV-MP12. Beads were washed to remove unbound virus and treated as indicated with 1) lysis buffer and wash, 2) pH 5.2 treatment followed by lysis buffer and wash, or 3) no pH 5.2 treatment and no lysis buffer. Protein bound to the avidin beads were resolved by SDS-PAGE and probed with the anti-RVFV Gc antibody 4D4. Data represent at least 3 separate experiments.

Like many enveloped viruses, the RVFV genome gains entry to a host cell's cytosol through a pH-dependent fusion of viral and host cell membranes [10]. To determine if the RVFV-6 peptide binds to RVFV at either neutral or low pH conditions such as would be observed during endocytosis, we performed immune-precipitation assays of RVFV-MP12 using biotinylated peptides bound to streptavidin beads. After washing, bound proteins were resolved by SDS-PAGE, and western blots were probed with a monoclonal antibody to Gc. When the immune precipitations were carried out at neutral pH, RVFV-6 and to a lesser extent RVFV-6sc, were found to precipitate Gc (Figure 5); however, in the presence of the non-ionic detergent Triton-X, which will solubilize the viral membrane, Gc was not precipitated. These results suggest that RVFV-6 did not bind to Gc directly. In contrast, when the same experiment was performed at low pH (pH 5.2), which is expected to trigger the Gc fusion mechanism, Gc was detected in the presence of Triton X (Figure 5). A lesser amount of binding was observed with the scrambled peptide, possibly due to the hydrophobic nature of the amino acids, suggesting that the amino acid order of the peptide by itself plays a role in the binding. As described in the Discussion, our data suggest a two-step mechanism for fusion inhibition like that reported earlier for DENV [18] in which the RVFV-6 peptide associates with the virion independent of Gc initially (e.g., binds to the viral membrane) but then specifically binds to Gc following the rearrangement in Gc triggered by the low pH treatment.

### RVFV-6 inhibits RVFV and VSV fusion

**Figure 6:**
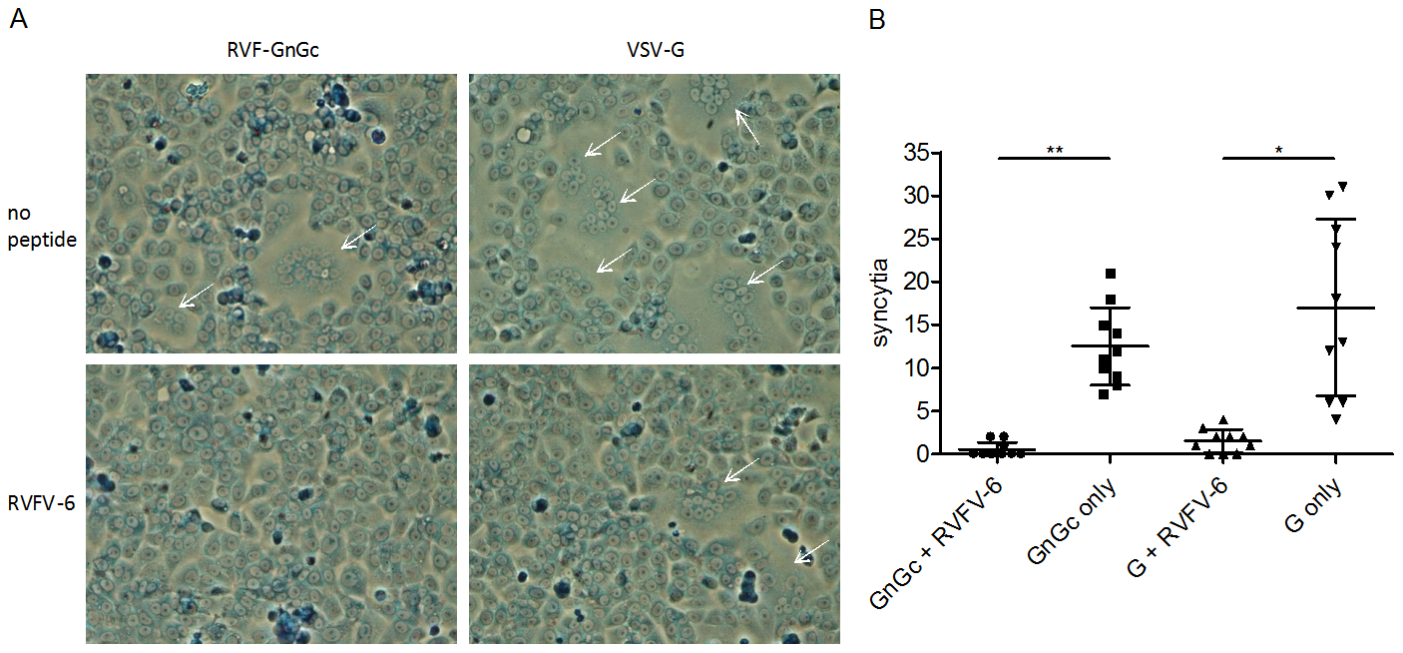
RVFV-6 inhibits both RVFV and VSV cell:cell fusion. Vero E6 cells were transfected with an expression plasmid expressing either the RVFV GnGc or VSV G. Twenty-four h later, cells were harvested and seeded into chamber slides. Eighteen h later, cells were incubated for 1 h with 50 µM RVFV-6 peptide followed by a pH 5.2 treatment. Medium was added to raise the pH, and slides were incubated for 5 h prior to methanol fixing and Giemsa staining. Fusion events (indicated with arrows) are shown in (A) and were quantified by number of syncytia per field of view at 100× in (B). Statistical significance was assessed by a paired, two-tailed t test. * (p = 0.001); ** (p<0.0001). Results are representative of at least 3 experiments.

Because RVFV-6 binds to Gc following low pH-induced structural rearrangements, we expected that its mechanism of action was indeed inhibition of viral fusion. To confirm this, we developed cell-cell fusion assays for both RVFV and VSV in which Vero E6 cells were transfected with plasmids expressing either the genes for RVFV GnGc or for VSV G. When these transfected cells were subjected to low pH treatment, the cells fused, forming syncytia (Figure 6A). However, if the cells were incubated with RVFV-6 and then subjected to low pH, the cell-cell fusion was significantly inhibited for both RVFV (p<0.0001) and VSV (p = 0.001) transfected cells (Figure 6A and B).

### Structural modeling

To gain some insight into the mechanism by which RVFV-6 is able to inhibit fusion processes mediated by three classes of fusion proteins, we applied molecular modeling techniques. As explained in Materials and Methods, full models for the fusion trimers of VSV G (Figure S2), EBOV GP2 (Figure S3), and RVFV Gc (Figure S4) were built combining homology modeling, fold recognition, and *ab initio* techniques. All three models show hydrophobic patches located near the fusion loops and at the interface between monomers that can serve as the stem binding sites. The content of aromatic residues at these sites is also high, consistent with the large number of aromatic residues in RVFV-6 and the identified stem regions in RVFV Gc, EBOV GP2, and VSV G proteins.

Since the RVFV-6sc peptide did unexpectedly bind to RVFV Gc in the immune-precipitation assay (Figure 5), structural analysis of the RVFV-6sc was also conducted (Figure S5). While the amino acids for this peptide were selected at random, the aromatic peptides of RVFV-6sc were distributed across the peptide such that there was an aromatic face (though not clustered at one terminus as with RVFV-6) that could still be capable of interacting with the hydrophobic groove exposed in the fusion protein during low-pH mediated conformational rearrangements. More of the hydrophobic residues (colored grey) are on the hydrophilic face of the peptide, and this could negatively impact membrane binding (reflected in Figures 4 and 5). This decreased membrane binding strength would have impacted the scrambled peptide's availability in the endosomal compartment and would explain the limited impact on infectivity.

## Discussion

In this report, we showed that a peptide (RVFV-6) analogous to the stem region of the putative fusion protein Gc of RVFV is capable of inhibiting multiple, diverse viruses in addition to RVFV. We conducted a series of studies to determine RVFV-6's mechanism of action. We demonstrated that the peptide 1) does not prevent virion binding to permissive cells; 2) binds to cells and virions independent of fusion protein binding; 3) binds to Gc following acidification; and 4) prevents viral fusion.

**Figure 7:**
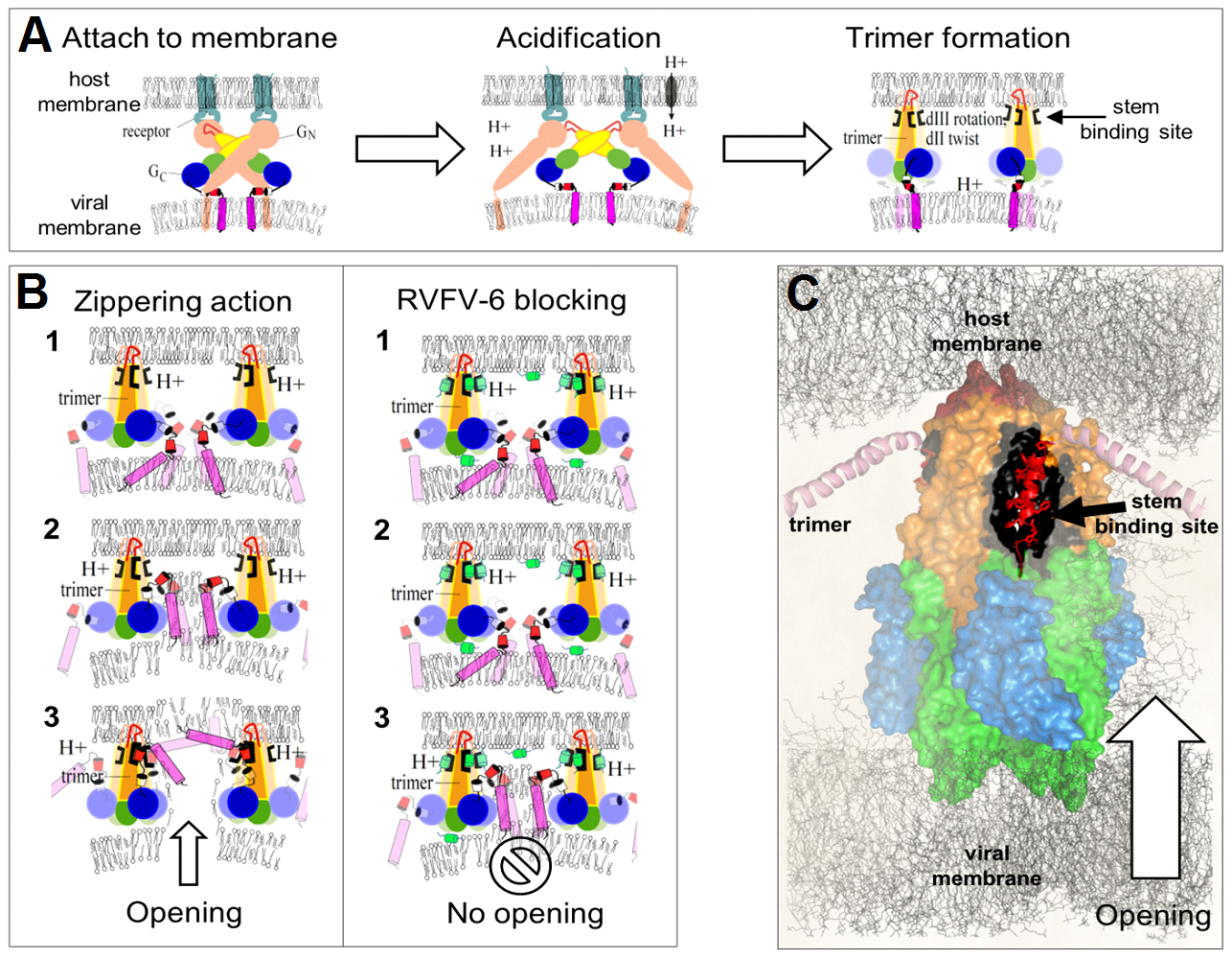
Molecular hypothesis of RVFV-6 mechanism of action. Panel A shows the initial stages of the membrane fusion process in bunyaviruses (adapted from [8]). Receptor-binding triggers uptake of virions by endocytosis. Acidification of the endocytic vesicle likely initiates Gn/Gc dissociation. Conformational rearrangements of domains I and II in Gc lead to trimer formation and insertion of the fusion peptide into the endosomal vesicle membrane. Trimer formation exposes the stem binding sites (shown in black) with affinity for the Gc stem and RVFV-6. The main molecular elements involved are: (*I*) glycoprotein Gc having four main components including [*a*] domains DI, DII, and DIII shown in green, orange/yellow, and blue, respectively; [*b*] the fusion loop shown in red; [*c*] the stem shown as small red cylinders; and [*d*] the transmembrane domains shown as a magenta cylinder; (*II*) the glycoprotein Gn, depicted as the receptor-binding protein of bunyaviruses, colored pink; and (*III*) host-cell receptors, shown in light-blue. Panel B: *Zippering action:* After acidification of the endocytic vesicle, the stem regions relocate, moving towards the host membrane through a zippering reaction. Docking of the stems into the stem binding sites is an essential step of membrane fusion that leads to opening of a pore or channel. *RVFV-6 blocking:* RVFV-6 molecules, shown as green cylinders, outcompete the stem fragment and lead to inhibition of fusion by blocking the movement of the native stem. Panel C: Molecular model of the RVFV Gc trimer complex. A molecular surface is used to highlight the three domains of the protein (colored as indicated in A). The stem fragments are shown as a red α-helices docked into the stem binding sites (black surface areas), and stem residues are highlighted using a stick representation.

Based on these findings and those of others, we propose that RVFV-6 prevents infection by first attaching to the viral and cellular membranes (see Figure 7A–C), likely due to interaction between the hydrophobic and aromatic residues of the peptide and the viral/cellular membranes. This binding would serve to both concentrate the peptide at the initial site of infection and also permit it to be internalized in concert with the virion. After internalization, the low pH environment of the endosome triggers a conformational rearrangement of Gc that exposes a previously hidden stem domain that is thought to interact with a “groove” on the low pH form of the glycoprotein, facilitating apposition of the virion and cell membranes. RVFV-6, analogous to the RVFV stem, may interact with this groove, preventing insertion of the stem and thereby blocking interactions between the virion and cell membranes (Figure 7B–C). A similar multistep mechanism has previously been proposed for potent peptides derived from the DENV E stem [48]. This stem-based mechanism is not universal, however, as one stem-based peptide inhibitor of DENV, DN59, can induce the formation of holes in the viral membrane, triggering premature release of the genome that causes the viral particles to become non-infectious even before interacting with cells [49]. Several interesting questions remain, including where on the fusion protein RVFV-6 binds and if the peptide's binding is reversible.

Because we designed peptides specifically against the RVFV stem region, we expected the fusion peptides to only inhibit RVFV or closely related viruses by binding to the complementary region in domain II of the fusion protein during low pH-mediated rearrangement. Thus, we were initially surprised to discover that RVFV-6 not only inhibited RVFV infection but also inhibited infection of other viruses including an unrelated bunyavirus (ANDV), a filovirus (EBOV) which uses a class I fusion protein, and a rhabdovirus (VSV) using a class III fusion protein. Structural modeling incorporating known features of various viral fusion proteins provided clues as to why this might occur: despite the differences in viral fusion protein class, all of the viral glycoproteins studied display similar structures in their membrane proximal regions (MPER) that can be modeled as highly amphipathic α-helices (Figure S5).

Our results with RVFV-6 inhibition are consistent with those from a recent mutagenesis analysis [50] which indicates that the stem region of flaviviruses interacts with a patch that forms a pocket in the DII domain of the E protein during initiation of the so-called zippering reaction. We believe that RVFV-6 can similarly interfere with such a zippering reaction, and we further postulate that such a mechanism (Figure 7) could be used by all the three classes of fusion proteins. We have generated 3-dimensional models of complete fusion trimers for RVFV, VSV, and EBOV in an attempt to identify the putative sites where RVFV-6 can dock. Supplementary Figures S2, S3, S4 highlight what we consider the most probable regions where the stems and the inhibitor RVFV-6 could bind in RVFV, VSV, and EBOV trimers. Therefore, it is possible that the RVFV-6 peptide binds to virion and cell membranes and traffics with the EBOV, ANDV, or VSV virions in endosomes in a similar manner as with RVFV. It is also possible that RVFV-6 could also block the functions of the structurally homologous regions of the ANDV, EBOV, and VSV fusion proteins following acidification and protein rearrangement, thus preventing successful fusion in the same manner as described for RVFV fusion (Figure 7). Indeed, we demonstrate that RVFV-6 does prevent VSV fusion using a cell:cell fusion assay. However, further experimental studies would be required to confirm our modeling and proposed mechanism, including determining if RVFV-6 does indeed bind to these viruses' fusion proteins following acidification and if RVFV-6 does prevent successful fusion of ANDV and EBOV. As such, we cannot rule out other potential mechanisms of action such as disruption and distortion of the viral or cellular membranes so that efficient viral entry is limited.

RVFV-6 did not show any inhibition of the alphaviruses VEEV, EEEV, or WEEV (which use class II fusion proteins) at high concentrations of peptide (50 µM). Similarly, the stem-based inhibitor DN59, which inhibits DENV and other flaviviruses [17], [49], did not inhibit the alphavirus Sindbis virus [17]. Taken together, these findings were initially confusing given the enhanced alphavirus inhibition observed when the stem is included with exogenous domain III [51] and the critical role the fusion protein stem plays in driving viral fusion of other viruses [11], [50]. Liao and Kielian found that, for the alphavirus Semliki Forest virus (SFV), the shorter (compared to flaviviruses) VFP stem had a minimal role in driving fusion but a critical role in virion assembly [52], suggesting that the domain III:trimer core interaction provides the primary force for SFV fusion. Conversely for flavivirus fusion, the stem:domain II groove interactions and zippering are crucial for efficient fusion [11], [50] and could be why stem-based peptides are capable of inhibiting viral infectivity.

In summary, we have identified a stem-based peptide that prevents viral fusion and infectivity by diverse viruses that utilize all three classes of viral fusion proteins. To our knowledge, this is the first description of a stem-based peptide that impacts infectivity of viruses that utilize three different classes of viral fusion proteins. Our findings are novel in that we have identified a potentially conserved feature of these three classes of fusion proteins that can be exploited for the development of broadly active antiviral fusion inhibitors. In addition, RVFV-6 and similar peptides could be used in competitive binding assays to identify broadly reactive small molecule drugs that could also block infectivity, expanding the utility of these peptides for therapeutics development.

## Supporting Information

Figure S1 **RVFV-6 binds to cells independent of RVFV fusion protein surface expression.** Vero E6 cells were incubated with biotin-conjugated RVFV-6 and stained with a gold-conjugated anti-biotin antibody. Transmission electron microscopy was conducted to visualize peptide location on the cell surface (arrows).
(TIF)

Figure S2 **Structural modeling of the VSV fusion trimer.** (A) Model of a complete fusion trimer of VSV generated from the available X-ray structure 2CMZ. The yellow box highlights the proposed docking site for the VSV stem. (B) Enhanced view of the region highlighted in panel A, showing a superposition of the RVFV-6 peptide and a fragment of the VSV stem. This putative docking site is generated during trimer formation and is compatible with RVFV-6 inhibition of VSV during fusion. Amino acids in the primary region of interest are shown using a ‘stick’ model and are colored according to their hydrophobicity, aromaticity, and charge: ALA, CYS, VAL, LEU, and MET are shown in white/gray scale; PHE, TYR, TRP, and PRO in a green scale; SER, GLN, and ASN in pink; GLU and ASP in red; and ARG, LYS, and HIS in blue/light-blue scale.
(TIF)

Figure S3 **Structural modeling of the complete EBOV fusion trimer.** This post-fusion model from EBOV Zaire was built using as templates (a) chains J, K, and N from the experimental structure of the pre-fusion trimer (PDB code: 3CSY) that contain the fusion loop fragments (fragments colored white), (b) the structure of post-fusion trimer (PDB code: 2EBO) that resolves the NHR (yellow) and CHR (green) fragments and the linker region (cyan), and (c) modeling *de novo* the C-terminal region of GP2 corresponding to residues 731 to 767 (residue numbers from NBCI sequence gi:|33860544|), colored pink with the stem fragment with high sequence similarity to RVFV-6 shown in red.
(TIF)

Figure S4 **Structural modeling of the RVFV Gc fusion trimer.** (A) Homology models for the RVFV Gc fusion trimer were generated using as templates the experimental structures of the glycoprotein Gc from RVFV (PDB code 4HJC), the homotrimer of the fusion glycoprotein E1 from Semliki Forest virus (PDB code: 1RER), and the structure of the E1 protein from VEEV (PDB code 3J0C-chain A). The fragments at the C-termini of the RVFV Gc fusion trimer were modeled *de novo* assuming that the stem region (highlighted in red) and trans-membrane fragments at the C-terminus both adopt α-helical conformations. (B) Enhanced view of the region containing the fusion loops (yellow box from panel A) showing the putative binding sites for the stem of RVFV (residues considered relevant are shown using a ‘stick’ representation and colored green). The putative docking sites are formed between DII domains after trimer formation.
(TIF)

Figure S5 **Structural modeling of viral stem regions for RVFV-6 inhibited virus, RVFV-6, and RVFV-6sc.** The sequences used in the comparison are: RVFV-6: WNFFDWFSGLMSWFGGPLK, RVFV-6sc – MFLGWSFDFGSLWGNKPWF, ANDV: FKCWFTKSGEWLLGILN, VSV: VELVEGWFSGWRSSLMGVLA, and EBOV: NWWTGWRQWIPAGIG. The atomic structures of the residues are shown using a CPK model. The following color code was applied: aromatic: black; hydrophobic: gray; weakly hydrophobic or neutral: white; acidic: red; basic: blue.
(TIF)
