# Supplementary figures and images for: A Fusion-Inhibiting Peptide against Rift Valley Fever Virus Inhibits Multiple, Diverse Viruses
Source: PLoS Negl Trop Dis. 2013 Sep 12;7(9):e2430. doi: 10.1371/journal.pntd.0002430 (PMC3772029; doi:10.1371/journal.pntd.0002430)

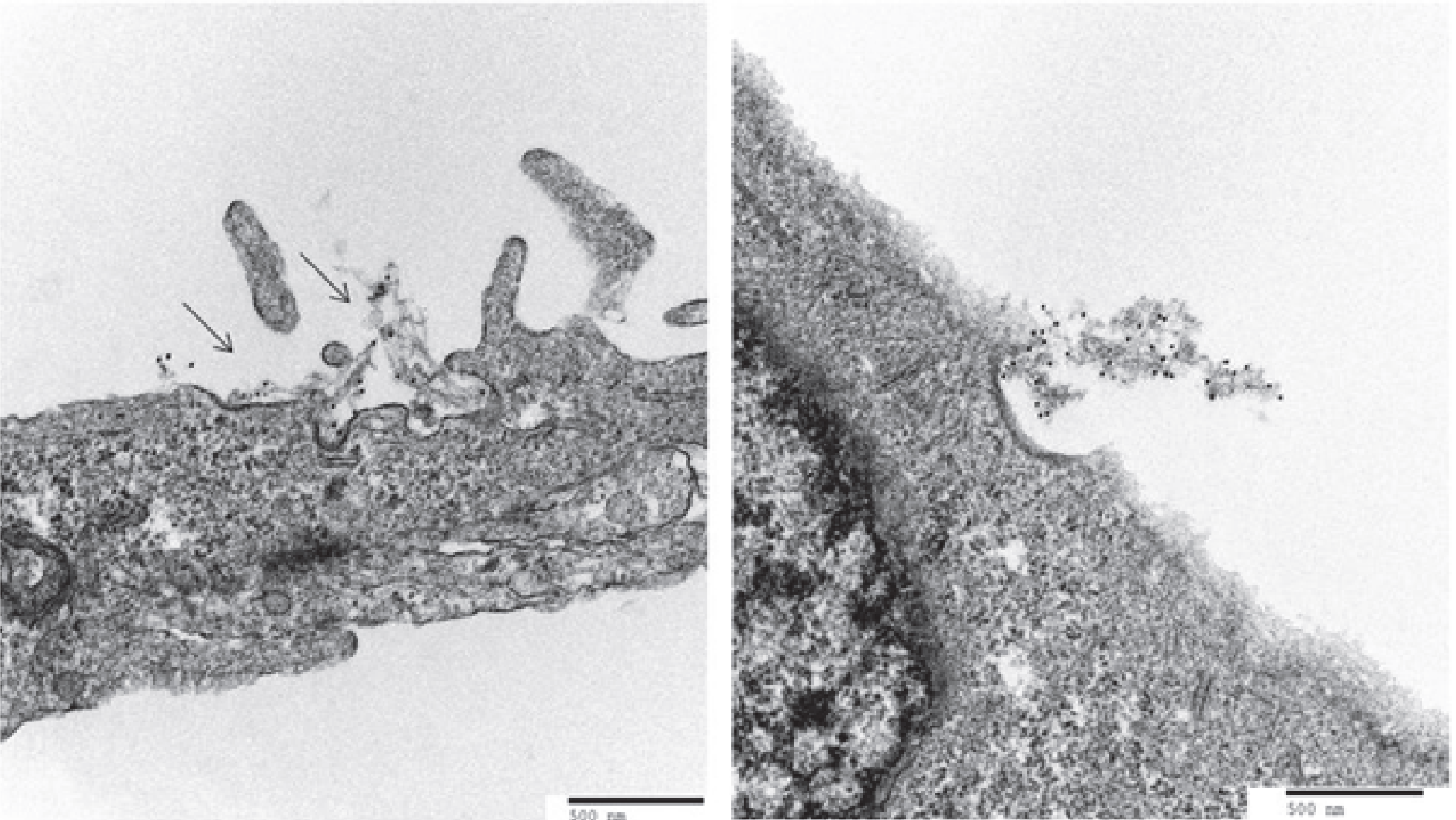

Supplement: Figure S1 — RVFV-6 binds to cells independent of RVFV fusion protein surface expression. Vero E6 cells were incubated with biotin-conjugated RVFV-6 and stained with a gold-conjugated anti-biotin antibody. Transmission electron microscopy was conducted to visualize peptide location on the cell surface (arrows). (TIF) [file pntd.0002430.s001.tif]

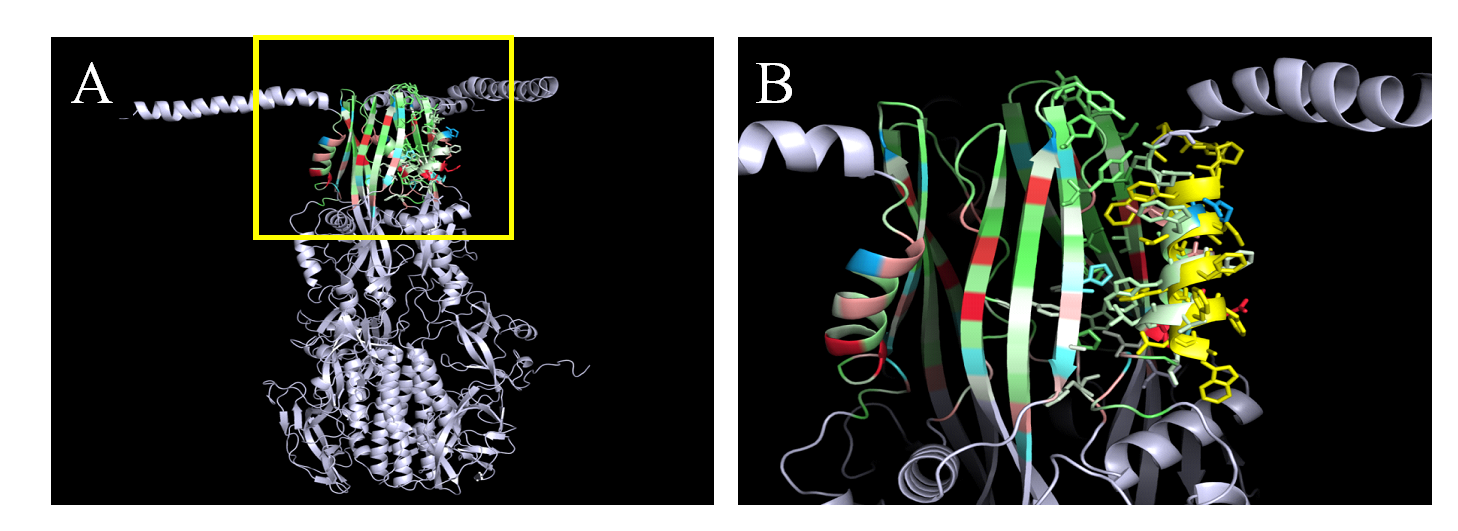

Supplement: Figure S2 — Structural modeling of the VSV fusion trimer. (A) Model of a complete fusion trimer of VSV generated from the available X-ray structure 2CMZ. The yellow box highlights the proposed docking site for the VSV stem. (B) Enhanced view of the region highlighted in panel A, showing a superposition of the RVFV-6 peptide and a fragment of the VSV stem. This putative docking site is generated during trimer formation and is compatible with RVFV-6 inhibition of VSV during fusion. Amino acids in the primary region of interest are shown using a ‘stick’ model and are colored according to their hydrophobicity, aromaticity, and charge: ALA, CYS, VAL, LEU, and MET are shown in white/gray scale; PHE, TYR, TRP, and PRO in a green scale; SER, GLN, and ASN in pink; GLU and ASP in red; and ARG, LYS, and HIS in blue/light-blue scale. (TIF) [file pntd.0002430.s002.tif]

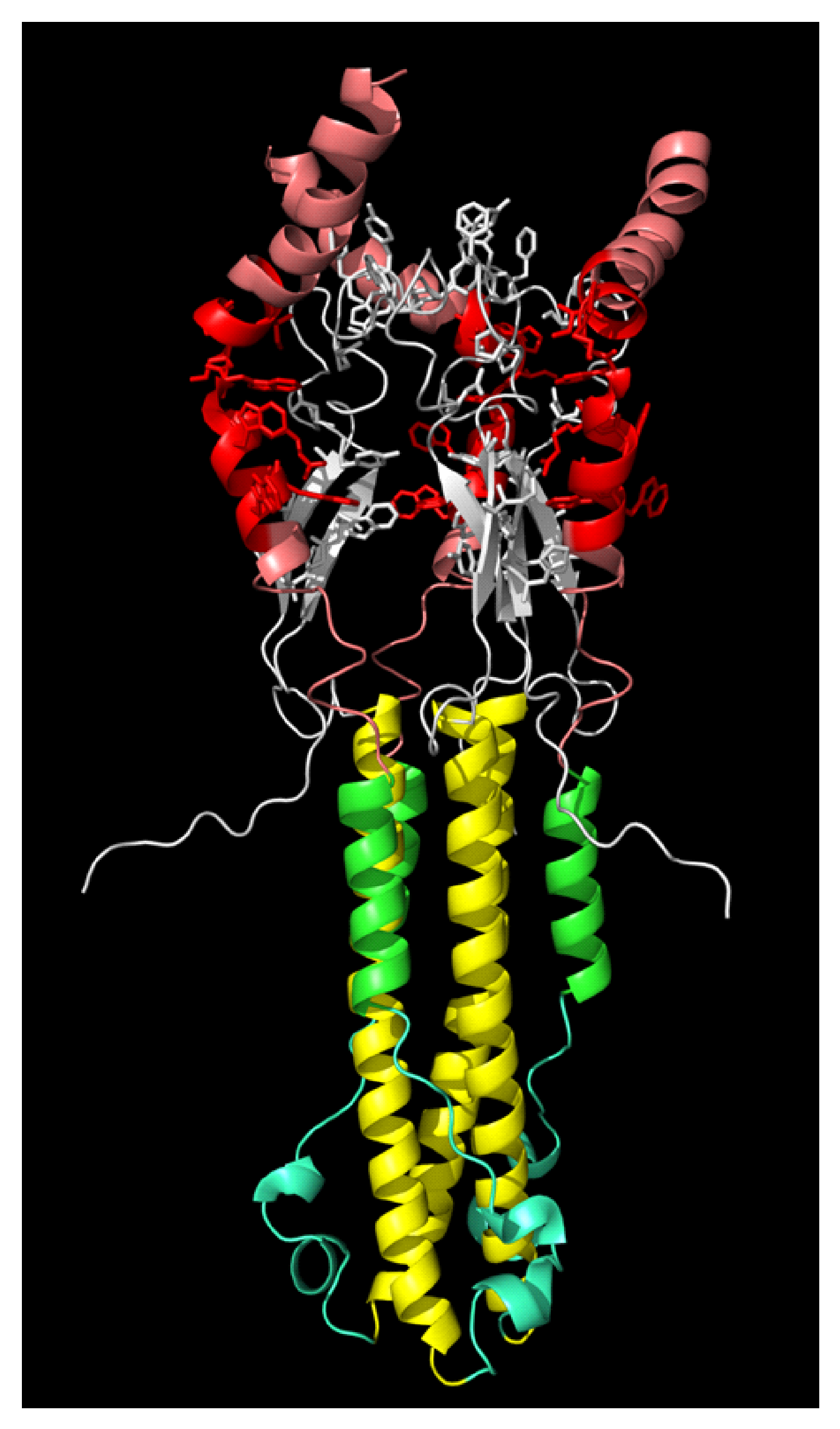

Supplement: Figure S3 — Structural modeling of the complete EBOV fusion trimer. This post-fusion model from EBOV Zaire was built using as templates (a) chains J, K, and N from the experimental structure of the pre-fusion trimer (PDB code: 3CSY) that contain the fusion loop fragments (fragments colored white), (b) the structure of post-fusion trimer (PDB code: 2EBO) that resolves the NHR (yellow) and CHR (green) fragments and the linker region (cyan), and (c) modeling de novo the C-terminal region of GP2 corresponding to residues 731 to 767 (residue numbers from NBCI sequence gi:|33860544|), colored pink with the stem fragment with high sequence similarity to RVFV-6 shown in red. (TIF) [file pntd.0002430.s003.tif]

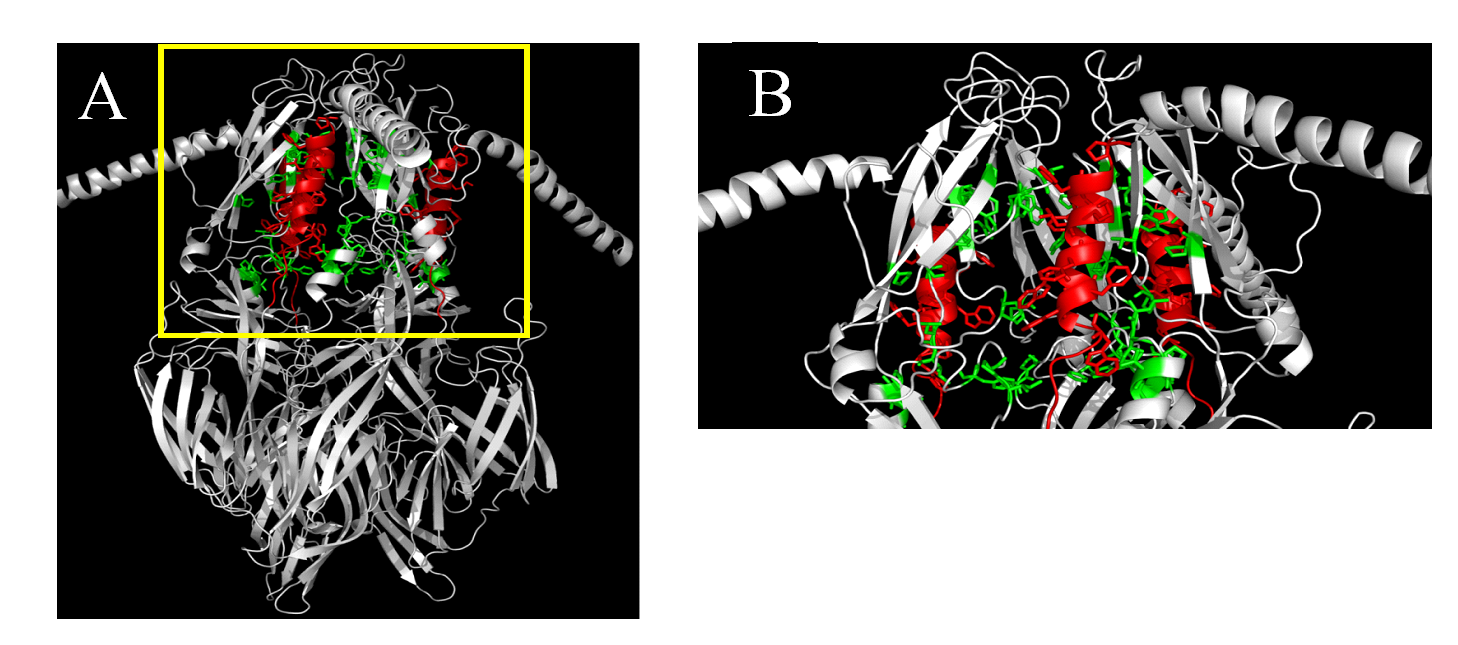

Supplement: Figure S4 — Structural modeling of the RVFV Gc fusion trimer. (A) Homology models for the RVFV Gc fusion trimer were generated using as templates the experimental structures of the glycoprotein Gc from RVFV (PDB code 4HJC), the homotrimer of the fusion glycoprotein E1 from Semliki Forest virus (PDB code: 1RER), and the structure of the E1 protein from VEEV (PDB code 3J0C-chain A). The fragments at the C-termini of the RVFV Gc fusion trimer were modeled de novo assuming that the stem region (highlighted in red) and trans-membrane fragments at the C-terminus both adopt α-helical conformations. (B) Enhanced view of the region containing the fusion loops (yellow box from panel A) showing the putative binding sites for the stem of RVFV (residues considered relevant are shown using a ‘stick’ representation and colored green). The putative docking sites are formed between DII domains after trimer formation. (TIF) [file pntd.0002430.s004.tif]

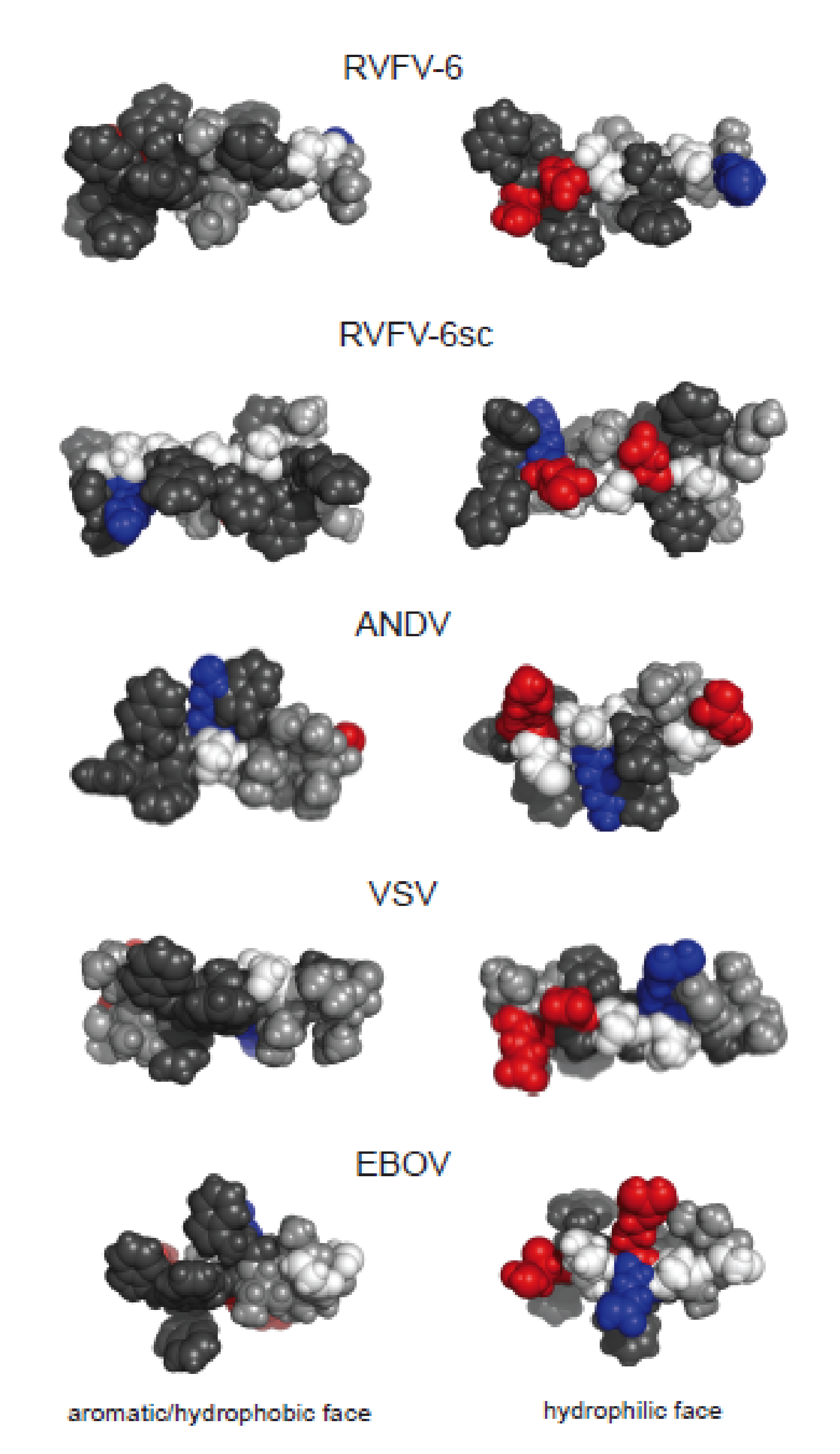

Supplement: Figure S5 — Structural modeling of viral stem regions for RVFV-6 inhibited virus, RVFV-6, and RVFV-6sc. The sequences used in the comparison are: RVFV-6: WNFFDWFSGLMSWFGGPLK, RVFV-6sc – MFLGWSFDFGSLWGNKPWF, ANDV: FKCWFTKSGEWLLGILN, VSV: VELVEGWFSGWRSSLMGVLA, and EBOV: NWWTGWRQWIPAGIG. The atomic structures of the residues are shown using a CPK model. The following color code was applied: aromatic: black; hydrophobic: gray; weakly hydrophobic or neutral: white; acidic: red; basic: blue. (TIF) [file pntd.0002430.s005.tif]
